# Differences in biomarker levels and proteomic survival prediction across two COVID-19 cohorts with distinct treatments

**DOI:** 10.1016/j.isci.2025.112046

**Published:** 2025-02-17

**Authors:** Cecilie Bo Hansen, Maria Elizabeth Engel Møller, Laura Pérez-Alós, Simone Bastrup Israelsen, Lylia Drici, Maud Eline Ottenheijm, Annelaura Bach Nielsen, Nicolai J. Wewer Albrechtsen, Thomas Benfield, Peter Garred

**Affiliations:** 1 Laboratory of Molecular Medicine, Department of Clinical Immunology, Section 7631, Copenhagen University Hospital - Rigshospitalet, Copenhagen, Denmark; 2 Department of Clinical Immunology, Copenhagen University Hospital - Rigshospitalet, Copenhagen, Denmark; 3 Department of Infectious Diseases, Copenhagen University Hospital - Amager and Hvidovre, Hvidovre, Denmark; 4 NNF Center for Protein Research, Faculty of Health and Medical Sciences, University of Copenhagen, Copenhagen, Denmark; 5 Department of Clinical Biochemistry, Copenhagen University Hospital - Bispebjerg Hospital, Copenhagen, Denmark; 6 Department of Clinical Medicine, Faculty of Health and Medical Sciences, University of Copenhagen, Copenhagen, Denmark

**Keywords:** Immunology, Proteomics

## Abstract

Prognostic biomarkers have been widely studied in COVID-19, but their levels may be influenced by treatment strategies. This study examined plasma biomarkers and proteomic survival prediction in two unvaccinated hospitalized COVID-19 cohorts receiving different treatments. In a derivation cohort (*n* = 126) from early 2020, we performed plasma proteomic profiling and evaluated innate and complement system immune markers. A proteomic model based on differentially expressed proteins predicted 30-day mortality with an area under the curve (AUC) of 0.81. The model was tested in a validation cohort (*n* = 80) from late 2020, where patients received remdesivir and dexamethasone, and performed with an AUC of 0.75. Biomarker levels varied considerably between cohorts, sometimes in opposite directions, highlighting the impact of treatment regimens on biomarker expression. These findings underscore the need to account for treatment effects when developing prognostic models, as treatment differences may limit their generalizability across populations.

## Highlights

• Proteomics-based mortality prediction in hospitalized COVID-19 cohorts
• Evaluation of innate immune markers, including several complement system components
• Prediction models show limited generalizability across cohorts
• Treatment regimes may influence prognostic capabilities of biomarkers

## Graphical abstract

**Figure:**
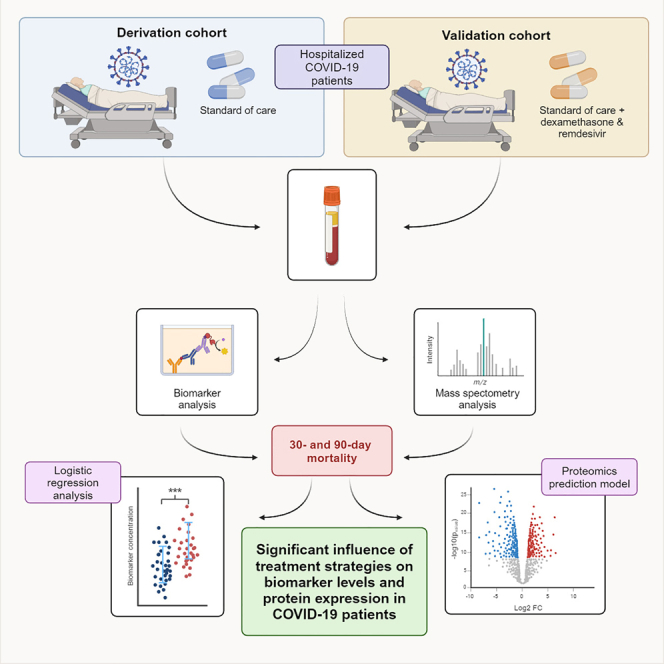

## Introduction

Throughout the Coronavirus disease 2019 (COVID-19) pandemic, tools to monitor disease progression, triaging, and predicting outcomes according to severity and high risk have been studied closely.^1^^,^^2^ Biomarkers are indicators of normal and pathological biological processes or responses to an exposure or intervention that can be objectively measured.^3^^,^^4^ Prognostic markers that can distinguish between different stages of disease, predict disease severity, or identify patients at risk of complications could significantly impact patient care and management.^4^ Several biomarkers and scoring systems have been shown to be of interest in COVID-19.^2^^,^^5^ However, the advantageousness of the markers varies according to the study cohorts.^1^ Several factors such as age, sex, severe acute respiratory syndrome coronavirus 2 (SARS-CoV-2) variant, treatment strategy, temporal trends, and comorbidities can differ between cohorts and hence influence the biomarker levels.^6^^,^^7^ Therefore, the impact and usefulness of the biomarkers should be confirmed in independent populations (validation cohorts). Validation cohorts are necessary to establish the reproducibility and generalizability of biomarker performance and assess the potential impact of population differences on biomarker validity. In this way, validation cohorts can help ensure that the biomarker performs consistently across diverse populations and settings, providing clinicians with reliable information for decision-making.

This study examined the robustness and utility of a panel of innate immune markers, including several complement system components, in two independent cohorts collected during the first and second waves of COVID-19 in Denmark receiving different treatment regimes. Furthermore, a random subset of the two cohorts was studied using plasma proteomics to establish a mortality prediction model in hospitalized patients with COVID-19.

## Results

The study included a derivation cohort (DC) (biomarker analysis: *n* = 126, proteomics analysis: *n* = 80) and a validation cohort (VC) (biomarker analysis: *n* = 112, proteomics analysis: *n* = 135) of patients with SARS-CoV-2 infection confirmed by reverse transcription–polymerase chain reaction (RT-PCR). All patients were admitted to Copenhagen University Hospital – Amager and Hvidovre in 2020. The selection of samples for each analysis (biomarker or proteomics) was random, driven by logistical factors and sample availability, with no specific samples chosen for either method. Consequently, differences in cohort size reflect sample availability at the time of analysis rather than any systematic selection process.

The baseline characteristics of the cohorts are presented in Table 1. The cohorts differed significantly regarding several parameters, including age, sex, comorbidity status, and mortality. The patients with DC were older, with a median age of 72 years [Interquartile range (IQR) 58–81] compared to 62 years [IQR 51–73] in the VC. The frequency of female patients was higher in the DC, with 73 females (58.0%) compared to 37 females (33.0%) in the VC. Furthermore, patients with DC more often had hypertension and cardiovascular disease. No difference was observed in the two cohorts' need for mechanical ventilation (MV) or extracorporeal membrane oxygenation (ECMO) treatment. However, 30-day mortality was significantly higher in the DC with 33 deaths (26.2%) compared to 15 deaths (13.4%) deaths in the VC (*p* = 0.016). This was also observed for 90-day mortality with 39 deaths (31.0%) in the DC compared to 16 deaths (14.3%) deaths in the VC (*p* = 0.003) (Table 1).

**Table 1:** Baseline demographic characteristics, treatment, clinical presentation, and outcome in the derivation and validation cohorts

Variables	Derivation cohort (*n* = 126)	Validation cohort (*n* = 112)	*p*
Age, years [IQR]	72 [58–81]	62 [51–73]	0.0003
Female, no (%)	73 (58.0)	37 (33.0)	0.0002
BMI, kg/m^2^ [IQR]	27.9 [24.2–32.0]^a^	29.1 [25.4–31.9]^b^	0.141

Comorbidity (%)	125 (99.2)	96 (85.7)	<0.0001

Hypertension, no (%)	60 (47.6)	34 (30.4)	<0.0001
Diabetes, no (%)	40 (31.7)	32 (28.6)	0.672
Cardiovascular disease, no (%)	71 (56.3)	29 (25.9)	<0.0001
Chronic lung disease, no (%)	12 (9.5)	7 (6.3)	0.474
Malignancy, no (%)	19 (15.1)	9 (8.0)	0.109
Other, no (%)	68 (54.0)	76 (67.9)	0.034

**Treatment**			

Remdesivir, no (%)	0 (0)	112 (100)	<0.0001
Dexamethasone, no (%)	0 (0)	107 (95.5)	<0.0001
Supplemental oxygen, no (%)	108 (87.1)^c^	110 (98.2)	0.001

**Clinical presentation**			

Radiographic evidence of pneumonic infiltration, no (%)	105 (83.3)	107 (95.5)	0.003
Days with symptoms [IQR]	7 [5–10]^d^	7 [5–9]^e^	0.202

**Outcomes**			

MV, no (%)	20 (15.9)	13 (11.6)	0.356
ECMO, no (%)	5 (4.0)	4 (3.6)	1.0
30-day mortality, no (%)	33 (26.2)	15 (13.4)	0.016
90-day mortality, no (%)	39 (31.0)	16 (14.3)	0.003

Values denote median [interquartile range] or number (%). MV = mechanical ventilation, ECMO = Extra Corporal Membran Oxygenation.

a Missing values = 18.

b Missing values = 14.

c Missing values = 2.

d Missing values = 28.

e Missing values = 6.

### Levels of biomarkers in the two cohorts

**Figure 1:**
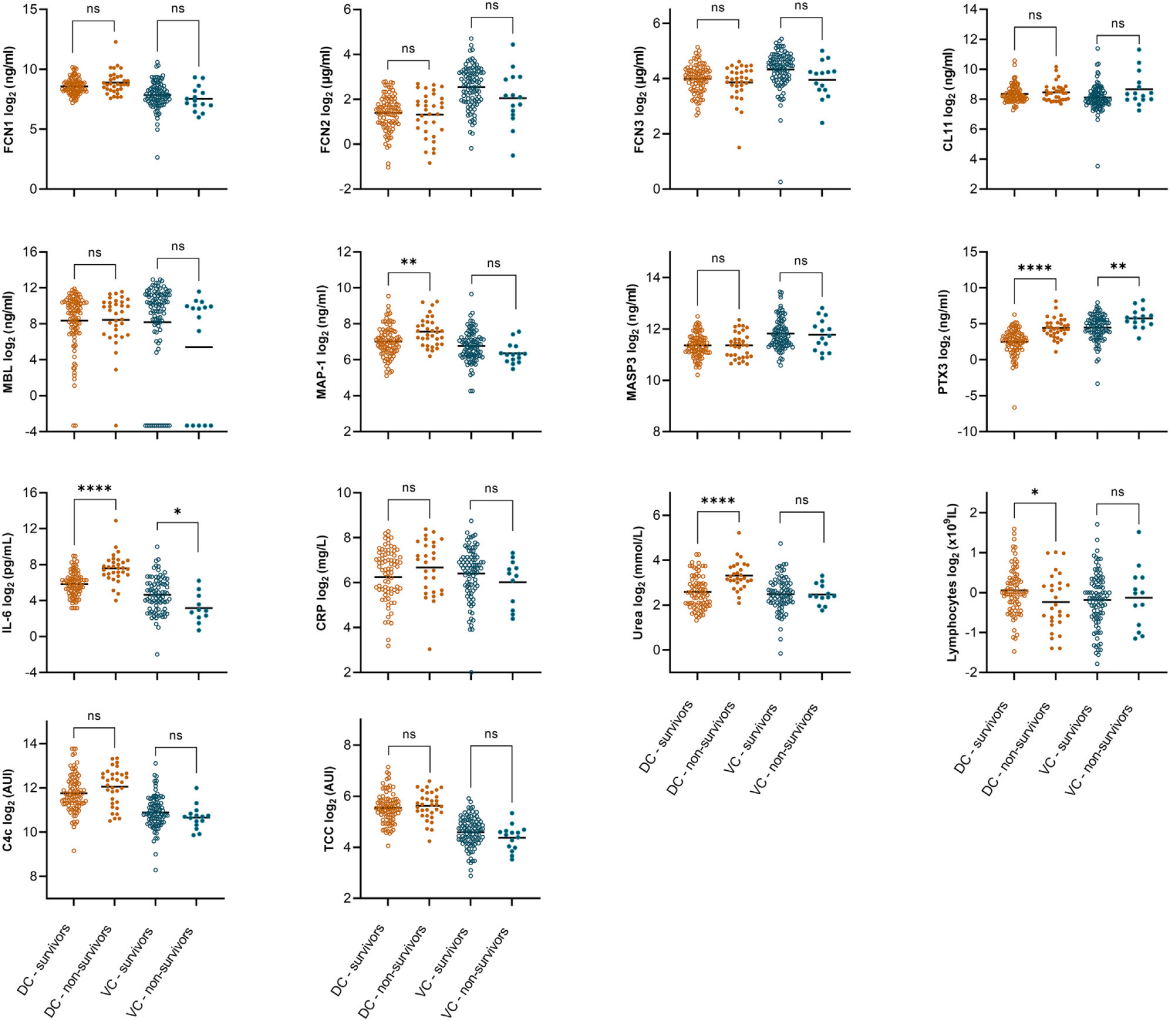
Biomarker levels Scatter dot plots of biomarker levels stratified by 30-day survival in the derivation cohort (DC) and validation cohort (VC). Yellow hollow dots represent DC survivors, yellow solid dots represent DC non-survivors, blue hollow dots represent VC survivors, and blue solid dots represent VC non-survivors. Horizontal black lines represent the mean. Differences between survivors and non-survivors were tested using an unpaired t-test. Non-significant, ns = *p* > 0.05; ∗ = *p* ≤ 0.05; ∗∗ = *p* ≤ 0.01; ∗∗∗ = *p* ≤ 0.001; ∗∗∗∗ = *p* ≤ 0.0001.

Biomarker levels upon admission to the hospital, divided according to 30-day mortality in the DC and VC, are presented in Figure 1. No significant differences between non-survivors and survivors in the two cohorts were observed for ficolin-1, ficolin-2, ficolin-3, collectin-11, mannose-binding lectin (MBL), mannose-binding lectin-associated serine protease-3 (MASP-3), C-reactive protein (CRP), C4c, and terminal complement complex (TCC). Long pentraxin 3 (PTX3) levels were significantly higher in non-survivors in both cohorts. Interleukin 6 (IL-6) levels were higher in non-survivors in the DC; however, the levels were significantly lower in non-survivors in the VC. MBL/Ficolin/Collectin-associated Protein-1 (MAP-1) and urea levels were higher in non-survivors in the DC, but no significant differences were observed in the VC. Lymphocyte count was lower in non-survivors in the DC, while no significant difference between survivors and non-survivors was observed in the VC. To assess the potential impact of selected demographic variables on biomarker levels in the DC and VC, all biomarkers were independently analyzed based on hypertension status (Figure S1), cardiovascular disease status (Figure S2), and sex (Figure S3). Additionally, correlations between age and biomarker levels were examined (Table S1).

**Figure 2:**
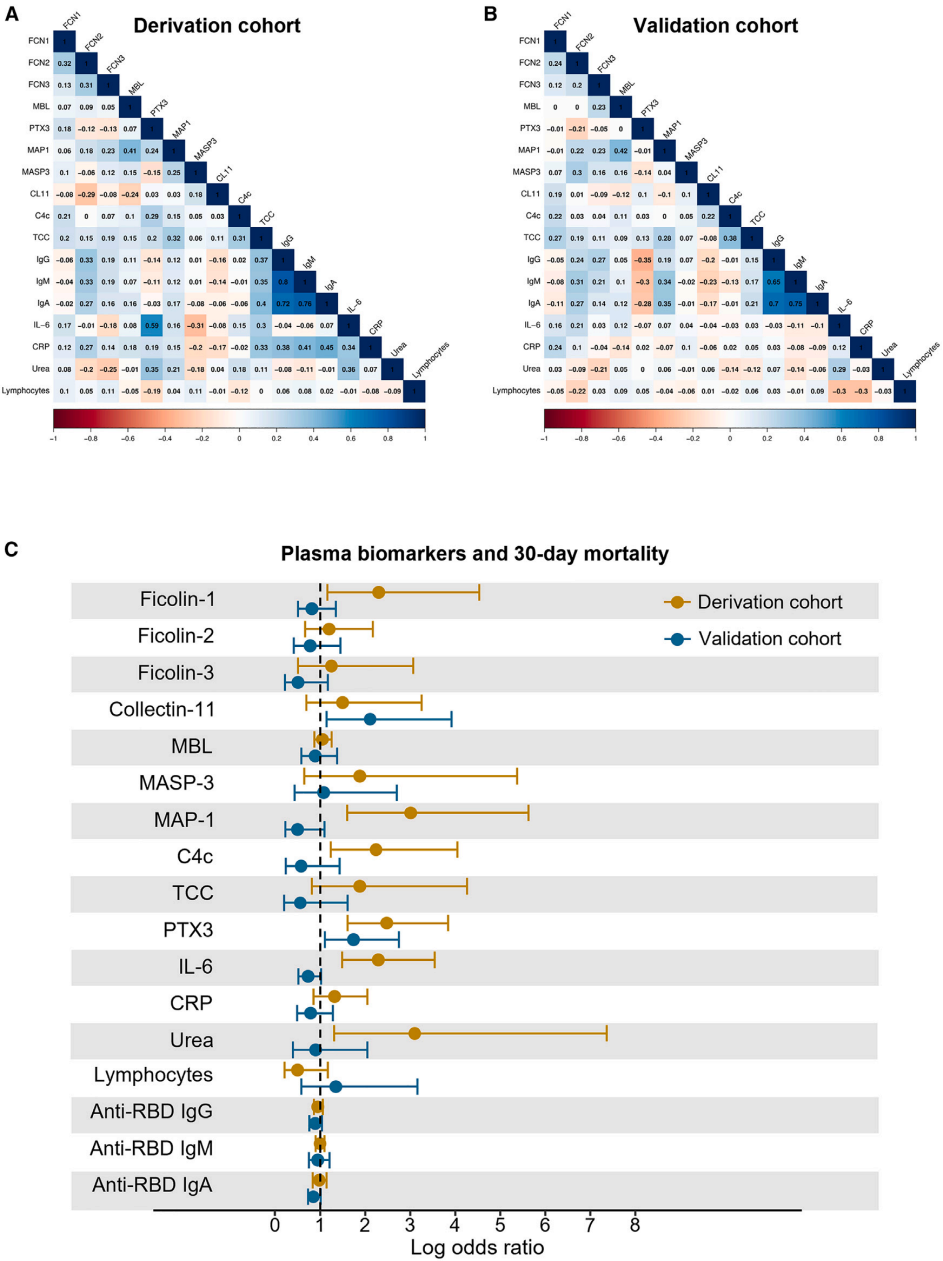
Correlation analysis and forest plot (A) Spearman correlation between plasma biomarkers in the derivation cohort (DC). (B) Spearman correlation between plasma biomarkers in the validation cohort (VC). (C) Forest plot displaying the adjusted logistic regression model results on biomarker association with 30-day mortality in the DC (yellow) and VC (blue). Dots represent values of log odds ratios and lines represent a 95% confidence interval.

Correlation analyses between the biomarkers illustrate discrepancies in associations in the two cohorts (Figures 2A and 2B). PTX3 and IL-6 were positively correlated in the DC (ρ = 0.59, *p* < 0.0001). However, no correlation was observed in the VC (ρ = −0.07, *p* = 0.534).

### Logistic regression – biomarkers and short-term mortality

Logistic regression analyses in relation to 30-day mortality were performed on the DC and VC. The adjusted model included age, sex, hypertension, and cardiovascular disease. Figure 2C depicts a forest plot of the adjusted odds ratios (ORs) of 30-day mortality per doubling of biomarker level. The unadjusted and adjusted ORs in relation to 30- and 90-day mortality in both cohorts are presented in Tables S2 and S3, respectively. A change in the direction of the OR between the DC and VC for several biomarkers was observed, with a tendency of increased odds in the DC and no or decreased odds in the VC (Figure 2C). This was particularly evident concerning MAP-1 and IL-6, and to a lesser extent ficolin-1, MASP-3, C4c, and urea levels. Each doubling of MAP-1 was associated with an OR of 3.01 (95% confidence interval [CI]: 1.60–5.63, *p* < 0.001) for 30-day mortality in the DC. However, in the VC each doubling of MAP-1 was associated with an OR of 0.52 (95% CI: 0.24–1.10, *p* = 0.087) for 30-day mortality. A doubling of IL-6 was associated with an OR of 2.29 (95% CI: 1.49–3.54, *p* < 0.001) in the DC and 0.73 (95% CI: 0.52–1.02, *p* = 0.062) in the VC. As previously reported,^8^ PTX3 was significantly associated with mortality in both cohorts.

### Proteomics analysis

**Figure 3:**
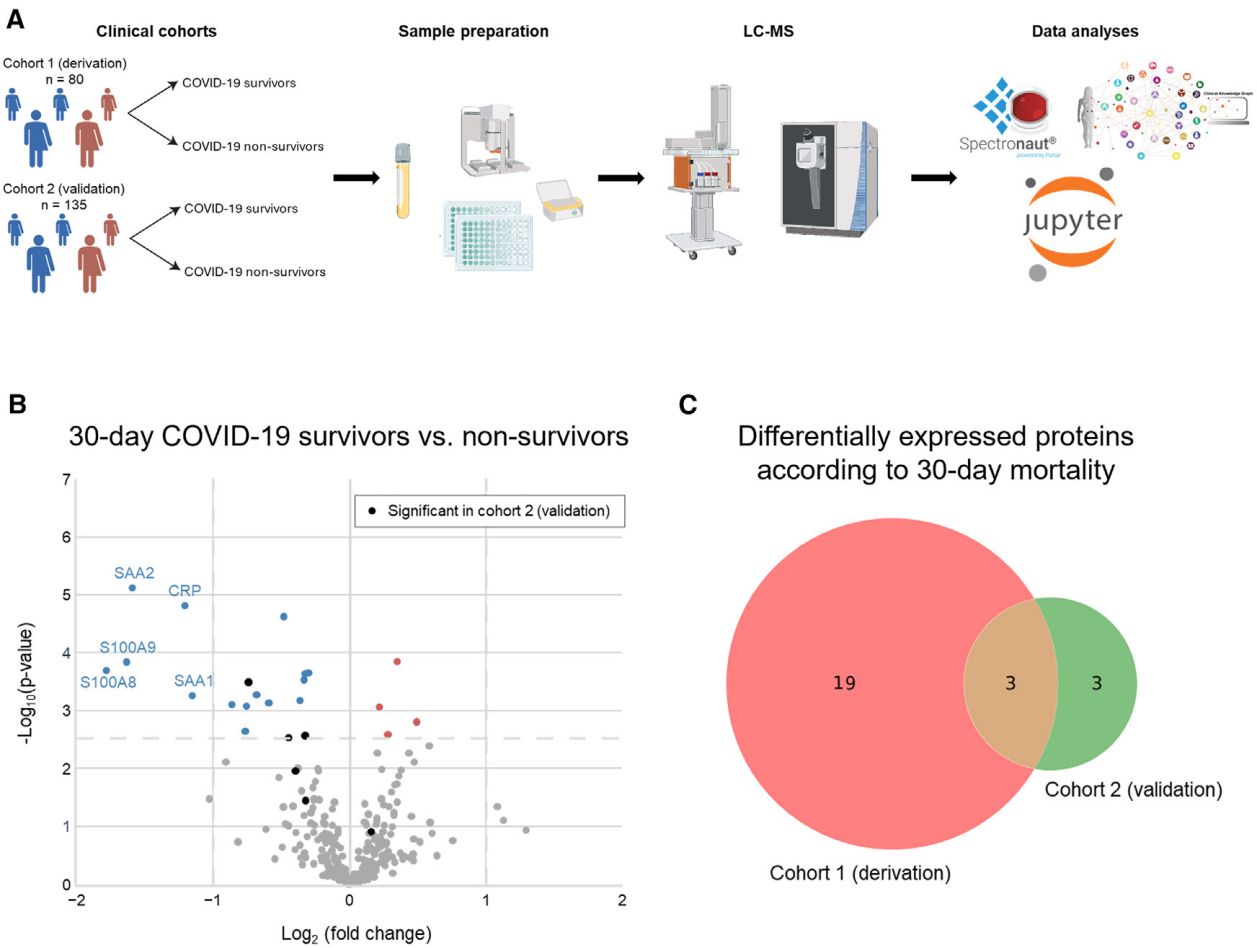
Proteomics analysis (A) Overview of proteomics study design. (B) Volcano plot depicting the fold change and associated *p*-values of proteins in the derivation cohort (cohort 1) in 30-day survivors versus non-survivors. Significant DEPs in the validation cohort (cohort 2) are presented as black dots. (C) Figure showing the number and overlap of DEPs between cohort 1 and cohort 2 according to 30-day mortality. The three overlapping DEPs: beta 2-microglobulin (B2M), leucine-rich alpha-2-glycoprotein 1 (LRG1), and prolidase or peptidase D (PEPD).

A liquid chromatography and mass spectrometry (LC-MS) analysis on a random subset of plasma samples from the DC and VC was performed to illustrate the distinction between the two cohorts further. Cohort 1 consisted of 80 samples from the DC collected in the spring of 2020 and cohort 2 consisted of 135 samples from the VC collected in the autumn/winter 2020 (Figure 3A). The baseline characteristics of the proteomics cohorts are presented in Table 2. Signatures of COVID-19 severity were identified by analyzing plasma proteins that underwent significant fold changes (FCs) in 30- and 90-day survivors and non-survivors. The data from the proteomics analysis of cohort 1 on 30-day mortality showed 18 downregulated and 4 upregulated proteins in survivors compared to non-survivors (Figure 3B). In cohort 2, the analysis revealed 5 downregulated and 1 upregulated protein in 30-day survivors versus non-survivors. In total, 3 differentially expressed proteins (DEPs) overlapped between the cohorts: beta 2-microglobulin (B2M), leucine-rich alpha-2-glycoprotein 1 (LRG1), and prolidase or peptidase D (PEPD) (Figure 3C). Proteomics analysis of cohort 1 on 90-day mortality showed 17 downregulated and 6 upregulated proteins in survivors compared to non-survivors (Figure S4A). Between the 30- and 90-day mortality timepoints 18 DEPs overlapped, and 9 DEPs differed. DEPs uniquely expressed by the 30-day timepoint included insulin such as growth factor binding protein acid labile subunit (IGFALS), von Willebrand factor (VWF), serum amyloid P-component (APCS), and PEPD. DEPs uniquely expressed by the 90-day timepoint included alpha-2-HS-glycoprotein (AHSG), carboxypeptidase N subunit 2 (CPN2), inter-alpha-trypsin inhibitor heavy chain H1 (ITIH1), peptidoglycan recognition protein 2 (PGLYRP2), and protein Z-dependent protease inhibitor (SERPINA10).

**Table 2:** Baseline demographic characteristics, treatment, clinical presentation, and outcome in the proteomics cohorts

Variables	Cohort 1 (Derivation cohort) (*n* = 80)	Cohort 2 (Validation cohort) (*n* = 135)	*p*
Age, years [IQR]	72 [55–80]	63 [50–74]	0.007
Female, no (%)	50 (62.5)	47 (34.8)	<0.0001
BMI, kg/m^2^ [IQR]	29.0 [24.6–32.2]^a^	28.7 [25.3–31.5]^b^	0.926
Comorbidity (%)	80 (100)	115 (85.2)	<0.0001
Hypertension, no (%)	38 (47.5)	41 (30.4)	0.013
Diabetes, no (%)	23 (28,8)	40 (29.6)	0.891
Cardiovascular disease, no (%)	44 (55.0)	36 (26.7)	<0.0001
Chronic lung disease, no (%)	9 (11.3)	8 (5.9)	0.194
Malignancy, no (%)	12 (15.0)	11 (8.2)	0.169
Other, no (%)	48 (60.0)	92 (68.2)	0.239

**Treatment**			

Remdesivir, no (%)	0 (0)	121 (89.6)	<0.0001
Dexamethasone, no (%)	0 (0)	118 (87.4)	<0.0001
Supplemental oxygen, no (%)	68 (86.1)^c^	125 (92.6)	0.154

**Clinical presentation**			

Radiographic evidence of pneumonic infiltration, no (%)	66 (82.5)	122 (90.4)	0.135
Days with symptoms [IQR]	8 [5–12]^d^	7 [4–9]^e^	0.017

**Outcomes**			

MV, no (%)	15 (18.8)	13 (9.6)	0.062
ECMO, no (%)	4 (5.0)	4 (3.0)	0.474
30-day mortality, no (%)	16 (20.0)	17 (12.6)	0.172
90-day mortality, no (%)	20 (25.0)	19 (14.1)	0.066

Values denote median [interquartile range] or number (%). MV = mechanical ventilation, ECMO = Extra Corporal Membran Oxygenation.

a Missing values = 13.

b Missing values = 17.

c Missing values = 1.

d Missing values = 15.

e Missing values = 6.

The 30-day mortality DEPs from cohort 1 were then subjected to Gene Ontology biological process (GOBP) enrichment and showed downregulation in processes related to leukocyte migration, neutrophil aggregation and chemotaxis, acute-phase and inflammatory responses in survivors vs. non-survivors (Figure S5).

### Prediction model

**Figure 4:**
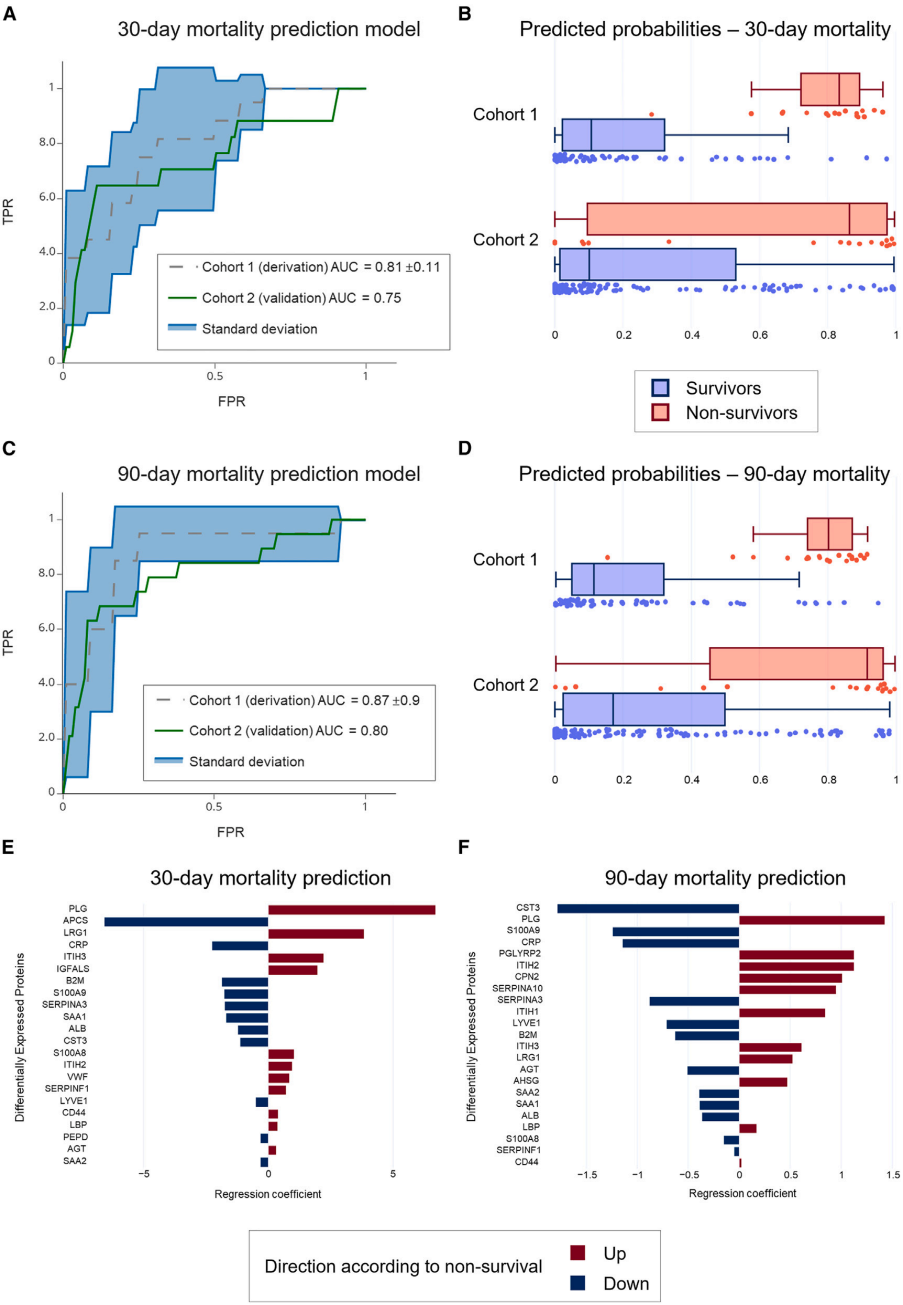
Proteomics prediction model (A) Receiver operating characteristic (ROC) curve analysis of the logistic regression prediction model trained on cohort 1 (derivation) and applied on cohort 2 (validation) for 30-day mortality. (B) Predicted probabilities of cohort 1 and cohort 2 according to 30-day survival (blue) and non-survival (red). (C) ROC curve analysis of the logistic regression prediction model trained on cohort 1 (derivation) and applied on cohort 2 (validation) for 90-day mortality. (D) Predicted probabilities of cohort 1 and cohort 2 according to 90-day survival (blue) and non-survival (red). (E) Plot illustrating the regression coefficients from DEPs of the 30-day prediction model and their up- (red) or down- (blue) regulation according to non-survival. (F) Plot illustrating the regression coefficients from DEPs of the 90-day prediction model and their up- (red) or down- (blue) regulation according to non-survival. down- (blue) regulation according to non-survival.

Using the LC-MS data generated from cohort 1, a prediction model according to 30- and 90-day mortality was established and trained. The 30-day mortality prediction model trained on cohort 1 showed a receiver operating characteristic (ROC) curve area under the curve (AUC) of 0.81 (Figure 4A). When applying the 30-day mortality prediction model in cohort 2 the model performed with a ROC curve of 0.75. The 90-day mortality prediction model was performed with an AUC of 0.87 in cohort 1 (Figure 4C). When using the 90-day mortality prediction model in cohort 2 the model performed with a ROC curve of 0.80. Plots of the predicted probabilities of 30- and 90-day survivors and non-survivors are shown in Figures 4B and 4D. Regression coefficients of the DEPs and their respective up- or downregulation according to survival from the prediction models of 30- and- 90-day mortality trained on cohort 1 (DC) are presented in Figures 4E and 4F.

## Discussion

The present study thoroughly examines the robustness and utility of a panel of innate immune molecules and proteins to predict mortality in two independent cohorts collected during the first and second waves of COVID-19 in Denmark. We find distinctive differences in biomarker levels between the cohorts depending on mortality, which could be related to the treatment strategy.

COVID-19 primarily presents as a respiratory infection but can develop into an inflammatory, multisystem disease involving an intricate interplay between the immune and coagulation systems, in which the dysregulated host response is involved in driving the pathology.^9^ Severe and critical COVID-19 is dominated by hyperinflammation, and the upregulation of several inflammatory biomarkers is indicative of clinical deterioration.^10^ In particular, complement activation and complement-related thromboinflammation have been associated with increased COVID-19 severity.^11^^,^^12^^,^^13^ Factors associated with a higher risk for severe COVID-19 include preexisting medical conditions such as malignancy, diabetes, chronic respiratory disease, and cardiovascular disease, but demographic factors such as age, sex, race, and ethnicity also play a role in disease severity.^14^^,^^15^^,^^16^^,^^17^^,^^18^

Although several inflammatory, hematological, and coagulation biomarkers have been shown to predict severity and mortality in COVID-19, temporal differences in SARS-CoV-2 variants, cohort demographics, and treatment strategies have changed throughout the pandemic and could consequently impact the robustness of these findings. In this study, we found discrepancies between the two study cohorts regarding several well-established biomarkers of severity and mortality, including IL-6 and CRP. Increasing IL-6 levels were associated with mortality in the DC, but surprisingly, we observed an inverse relationship in the VC. The DC sampling took place during the first wave of COVID-19, in the spring of 2020, when no standard treatment guideline was established in Denmark, and patients admitted to the hospital received thrombosis prophylaxis and oxygen therapy as deemed necessary by the treating physician. Patients with VC were included after the introduction of remdesivir and systemic corticosteroids as part of the standard treatment regimen along with thrombosis prophylaxis and oxygen therapy, and all included patients received remdesivir and 95.5% also received dexamethasone.

Corticosteroids, including dexamethasone, have broad anti-inflammatory and immunosuppressive effects.^19^^,^^20^ Reduction in complement activation levels (C3a and C5a) and the pro-inflammatory cytokines IL-1β, −6, and −8 has been demonstrated in patients receiving corticosteroids (methylprednisolone and/or dexamethasone) following cardiopulmonary bypass compared to controls illustrating the extensive anti-inflammatory effects of these therapeutics.^21^^,^^22^ Moreover, a reduction of IL-6 in hospitalized patients with COVID-19 following both remdesivir and corticosteroid administration has been reported.^23^ IL-6 receptor blockers (tocilizumab or sarilumab) are now recommended for treatment in severe and critical COVID-19, together with corticosteroids.^24^ Remdesivir is a direct-acting nucleotide prodrug inhibitor of the SARS-CoV-2 RNA-dependent RNA polymerase and treatment may lead to more rapid normalization of several biomarkers associated with COVID-19 severity, including ferritin, lactate dehydrogenase, prothrombin time, and lymphocyte count.^25^^,^^26^ With 95.5% of patients with VC receiving both dexamethasone and remdesivir before sampling, we suggest that the treatment strategy may influence biomarker levels, potentially affecting the ability of these biomarkers to predict mortality.

Complement system overactivation in COVID-19 with the increased production of complement components and activation products and increased consumption depending on disease severity have been reported extensively.^13^^,^^27^^,^^28^^,^^29^^,^^30^^,^^31^^,^^32^^,^^33^ In this study, increasing C4c levels were associated with mortality in the logistic regression analysis in the DC but not the VC. Terminal complement complex (TCC) levels were not associated with mortality in either cohort. Lectin pathway-associated molecules ficolin-1 and MAP-1 were associated with mortality in the DC, whereas collectin-11 was associated with mortality in the VC. Contradictory results have been reported regarding whether collectin-11 and ficolins can bind to the SARS-CoV-2 spike (S) protein and thereby activate the lectin pathway.^34^^,^^35^

Proteomic profiling of COVID-19 pathology and severity markers have been studied throughout the pandemic and several interesting markers have been characterized. We identified 22 DEPs between survivors and non-survivors in the DC (cohort 1) and 6 DEPs in the VC (cohort 2) according to 30-day mortality, with an overlap of 3 proteins between the cohorts. Several of the DEPs have been reported previously in relation to COVID-19 severity, including S100 calcium binding protein A8/A9 (S100A8/9), serum amyloid A1/A2 (SAA1/2), CRP, cystatin C (CST3), angiotensinogen (AGT), and ITIH2.^36^^,^^37^^,^^38^^,^^39^^,^^40^^,^^41^^,^^42^^,^^43^ To highlight some of the DEPs, S100A8/A9 also known as calprotectin when in heterodimeric complex, are abundant cytosolic proteins involved in the intracellular pathway regulation of inflammatory responses in various innate immune cells.^44^ Higher levels of S100A8/9 have been observed in severe and fatal patients with COVID-19 needing ICU-level management compared to patients with COVID-19 not requiring ICU admission.^45^ ITIH2 is part of the inter-α-trypsin inhibitor family, involved in extracellular matrix stability,^46^ and has been found to be more abundant in moderate/severe COVID-19 disease and survivors compared to critical disease and in non-survivors.^39^^,^^47^ AGT is a crucial part of the renin-angiotensin system (RAS), a key regulator of blood volume, electrolyte balance, and systemic vascular resistance, and is secreted by the liver and cleaved by renin to form angiotensin I.^48^ We found that AGT levels were higher in non-survivors in the DC (cohort 1). The association between high levels of AGT and increasing severity has been observed by others.^36^^,^^39^ Another central molecule of the RAS is angiotensin-converting enzyme 2 (ACE2), which is the host target receptor used by SARS-CoV-2 and SARS-CoV for cellular entry.^49^^,^^50^ Three DEPs were observed with decreased levels in survivors compared to non-survivors in both cohorts according to 30-day mortality: B2M, LRG1, and PEPD. B2M, a component of the major histocompatibility complex class I (MHC I)^51^ is involved in normal and pathological immune responses and inflammation.^52^ LRG1 is part of the family of leucine-rich repeat (LRR) proteins and a multifunctional pro-inflammatory signaling molecule upregulated in several diseases, including COVID-19.^53^ PEPD is a ubiquitously expressed cytosolic metalloproteinase involved in protein metabolism and collagen matrix remodeling, and has been shown to stimulate the expression and maturation of the interferon-I receptor and thereby impact type I interferon signaling.^54^^,^^55^ Following these observations, the GO biological process enrichment analysis showed downregulation in processes related to leukocyte migration, neutrophil aggregation and chemotaxis, and inflammatory responses in survivors vs. non-survivors.

Besides minor differences in the collection and expression of DEPs between the 30- and 90-day models, 9 DEPs differed between the two timepoints in the DC (cohort 1), our analysis revealed a notable enhancement in the ROC AUC when assessing 90-day mortality compared to the short-term 30-day mortality endpoint. The elevation in AUC (from 0.81 to 0.87) observed when extending the follow-up period suggests a pronounced improvement in predictive power, likely attributed to the increased representation of non-survivors (*n* = 4) in the dataset. However, we believe conclusions about the relevance of uniquely expressed proteins in predicting short- and long-term mortality would be highly speculative in the context of this study format.

As hyperinflammation, with the upregulation of a myriad of inflammatory biomarkers, is part of the disease progression in severe COVID-19, and a crucial part of the treatment strategy is broad immune suppression, the resulting levels of inflammatory biomarkers will be a complex mix of pro- and anti-inflammatory signaling. A plasma proteomics study showed that dexamethasone suppressed 10 host proteins previously identified as biomarkers discriminating severe from non-severe COVID-19 disease, including S100A8, S100A9, SERPINA1, SERPINA3, orosomucoid-1 (ORM1), lipopolysaccharide-binding protein (LBP), VWF, polymeric immunoglobulin receptor (PIGR), alpha-2-glycoprotein 1, zinc-binding (AZGP1), and CRP.^38^^,^^56^ We found several of these proteins (S100A8, S100A9, SERPINA3, LBP, and CRP) downregulated in survivors in the DC; however, this was not observed in the VC treated with dexamethasone. Dexamethasone has been shown to efficiently inhibit the SARS-CoV-2-induced *in vitro* expression of chemokines and cytokines in PBMCs at both the transcriptional and protein level.^57^ Therefore, the timing of sample collection in relation to disease progression and treatment strategy could have a big impact on the biomarker levels, adding complexity to data interpretation.^58^^,^^59^ We hypothesize that a significant part of the differences observed between the two cohorts in both the enzyme-linked immunosorbent assay (ELISA) measured biomarkers and the proteomics study is based on differences in the immunomodulatory treatment between the cohorts.

In conclusion, this study evaluates the robustness and utility of a panel of innate immune markers and a proteomics-based prediction model in two independent COVID-19 cohorts. We find distinct differences between the cohorts in biomarker levels, which could be attributed to the treatment strategy, as patients in the derivation cohort did not receive either remdesivir or dexamethasone. In contrast, the validation cohort patients received both treatments. Going forward, as many prognostic markers have been identified early on in the COVID-19 pandemic in cohorts sampled before the implementation of standardized treatment guidelines, the impact of specific treatments on biomarker levels and proteomic signatures should be carefully interpreted. This study highlights the significant influence of treatment strategies on biomarker levels in patients with COVID-19 and underscores the need for the careful consideration of treatment variables when developing prognostic models. The reduced generalizability of our predictive model in different cohorts points to the complex interplay between treatment regimens and biomarker expression, calling for context-specific approaches in prognostic biomarker research for COVID-19. We suggest a heightened focus on the impact of immunomodulators on biomarker expression, especially in diseases dominated by hyperinflammation, such as COVID-19, because this could both aid in defining optimal time for the initiation of treatment and secure more robust prognostic tools for clinical decision-making.

### Limitations of the study

Due to the nature of the observational study design the present work might be subject to potential bias due to unknown confounders and thereby limit conclusions regarding causality. We cannot state anything about temporal trends or dynamics of the different proteins investigated in the study as we do not have continuous sampling. It is a single-center study which limits generalizations of results and also restricts the number of study participants. As addressed above, the DC and VC differ notably regarding age distribution, sex distribution, comorbidities, and pharmacological treatment of the patients. Mass spectrometry based proteomics suffers from sensitivity challenges and proteins circulating at low concentrations may not be identified. All of these factors should be considered when interpreting the results. On the other hand, proteomics can offer valuable and unbiased information about disease pathology and progression, without prior knowledge about the specific biological pathways and molecules involved. Moreover, samples were collected within four days of hospital admission and the patients were followed prospectively.

## Resource availability

### Lead contact

Further information and requests for resources and reagents should be directed to and will be fulfilled by the lead contact, Cecilie Bo Hansen (cecilie.bo.hansen@regionh.dk).

### Materials availability

This study did not generate new unique reagents.

### Data and code availability

• The mass spectrometry proteomics data have been deposited to the ProteomeXchange Consortium via the PRIDE^60^ partner repository with the dataset identifier PXD055973.
• This article does not report the original code.
• Any additional information required to reanalyze the data reported in this article is available from the lead contact upon request.

## Acknowledgments

We thank Mss Bettina Eide Holm, Mads Engelhardt Knudsen, and Anna Louise Sørensen for excellent technical assistance. Furthermore, we thank Christine Rasmussen (Department of Clinical Biochemistry, Copenhagen University Hospital - Bispebjerg) for her time planning, preparing and the proteomic analysis, the Department of Clinical Biochemistry, Rigshospitalet, and the Clinical Proteomic Group at the NNF Center for Protein Research, University of Copenhagen, in particular Matthias Mann. We would also like to acknowledge the patients and staff at Copenhagen University Hospital – Amager and Hvidovre who made this study possible. This work was supported by grants from the 10.13039/501100002808Carlsberg Foundation (CF20-0045) and the 10.13039/501100009708Novo Nordisk Foundation (NFF205A0063505). Nicolai J. Wewer Albrechtsen received funding from the 10.13039/501100009708Novo Nordisk Foundation (NNF19OC0055001). 10.13039/501100024571Novo Nordisk Foundation Center for Protein Research (CPR) is supported financially by the 10.13039/501100009708Novo Nordisk Foundation (NNF14CC0001).

## Author contributions

C.B.H., N.J.W.A., T.B., and P.G. performed conceptualization and study design. C.B.H., M.E.E.M., L.P.-A, and S.B.I. LD performed the data collection. C.B.H., P.G., L.D., M.E.O., A.B.N., and T.B. performed the data analysis. C.B.H. wrote the first draft of the manuscript. All authors read and approved the final manuscript.

## Declaration of interests

The authors declare no competing interests.

## STAR★Methods

### Key resources table

**Table:** 

REAGENT or RESOURCE	SOURCE	IDENTIFIER
**Biological samples**		

Plasma samples from hospitalized COVID-19 patients	Department of Infectious Diseases, Copenhagen University Hospital - Amager and Hvidovre, Hvidovre	

**Deposited data**		

The mass spectrometry proteomics data	The PRoteomics IDEntifications (PRIDE) database (https://www.ebi.ac.uk/pride/)	PXD055973

**Software and algorithms**		

Spectronaut version 15	Biognosys	https://biognosys.com/resources/spectronaut-a-groundbreaking-increase-in-identifications/
GraphPad Prism version 9.3.1	GraphPad Software	https://www.graphpad.com/
R version 4.1.0	R Foundation for Statistical Computing	https://www.r-project.org/
Research Electronic Data Capture (REDCap) browser-based software	Harris et al.^61^	https://project-redcap.org/

### Experimental model and study participant details

The DC comprised adults aged 18 years or older admitted to Copenhagen University Hospital – Amager and Hvidovre with confirmed SARS-CoV-2 infection in the spring of 2020 (between March 10 and May 31). In-depth characteristics of the cohort have been described previously.^62^ In Denmark, no standardized treatment guideline was established at this point. Instead, DC patients received standard of care (SOC) including thrombosis prophylaxis and oxygen therapy as deemed necessary by the treating physician. The VC included adults aged 18 years or older admitted to Copenhagen University Hospital – Amager and Hvidovre in the autumn/winter of 2020 (between September 7 and December 14). Details of the VC have been described elsewhere.^63^ VC patients received SOC along with remdesivir and dexamethasone. In brief, consecutive cases admitted were included. All cases were confirmed by RT-PCR on an oropharyngeal swab or lower respiratory tract specimen. Data, including patient characteristics, clinical presentation, and medical history, were extracted from electronic health records and managed using Research Electronic Data Capture (REDCap) browser-based software.^61^ Sex and age were extracted from patients' unique civil registration numbers. Blood sampling was performed within four days of admission and the samples were separated by centrifugation and stored at minus 80°C. The study was approved by the Danish Patient Safety Authority (record no. 31-1521-309) and the Regional Data Protection Center (record no. P-2020-492). The Ethics Committee approved measurements of biomarkers in stored samples from the biobank of the Capital Region of Denmark (record no. H-20047597). The committee exempted a requirement of individual informed consent.

### Method details

#### Enzyme-linked immunosorbent assays

Ethylene Diamine Tetra Acetic acid (EDTA) plasma levels of the different complement components MBL, MAP-1, MASP-3, ficolin-1, ficolin-2, ficolin-3, collectin-11, PTX3, C4c, and TCC were quantified via specific sandwich ELISAs developed in the Laboratory of Molecular Medicine, Rigshospitalet, according to previously described methods.^64^^,^^65^^,^^66^^,^^67^^,^^68^^,^^69^^,^^70^^,^^71^^,^^72^^,^^73^ Levels of IgM, IgA, and IgG specific for the receptor-binding domain (RBD) of the SARS-CoV-2 ancestral strain S protein were determined using in-house ELISAs.^74^ All assays have been adapted to automated analysis in a 384-well format on the Biomek FX robotic system (Beckman Coulter, Fullerton, CA, USA).

#### IL-6 levels and routine biochemistry

IL-6 levels were determined according to the manufacturer’s instructions using magnetic fluorescently labeled microsphere beads (R&D Systems, Abingdon, UK) and analyzed on a BioPlex 200 (Bio-Rad). CRP, urea, and lymphocyte count were measured as part of routine sampling and analyzed by the Department of Clinical Biochemistry at Copenhagen University Hospital – Amager and Hvidovre.

#### Proteomics

##### Protein digestion and Evotips loading

Sample preparation was performed on an Agilent Bravo Liquid Handling Platform according to the previously published method described in Geyer et al.^75^ Briefly, plasma samples were aliquoted into a 96-well format plate and introduced to the Bravo Robot. Each plasma sample was diluted 1:10 with the PreOmics Lysis buffer (P.O. 00001, PreOmics GmbH) and 20 μL of the dilution was incubated at 95°C for 10 min in order to denature proteins, reduce disulfide bridges and alkylate cysteines Kulak et al.^76^ After cooling the sample for 15 min at room temperature, trypsin and LysC were added in a ratio of 1 μg enzyme to 100 μg proteins and the mixture was incubated at 37°C for 4 h. The enzymatic reaction was quenched by the addition of 64 μL of 0.2% TFA and the samples were loaded onto Evotips (Evosep Biosystem, Denmark) according to the manufacturer’s recommendations. The Evotips were wetted with isopropanol for 5 min, activated with 20 μL solvent B (99% ACN, 0.1% FA) and centrifuged at 700xg for 1 min 20 μL of buffer A was then added to equilibrate the tips followed by sample loading. Finally, 20 μL Buffer A was used to wash the Evotip and 100 μL was added to avoid drying.

##### LC-MS analysis

The samples were injected to an Exploris 480 Thermo Fischer Scientific system using an Evosep One instrument (Evosep Biosystem). A preset chromatographic method was used corresponding to 60 samples per day. The peptides were separated on an 8 cm Pepsep (Marslev, Denmark) column (100 μm ID/3 μm bead size Reprosil-Pur C18 beads) at 1 μL/min flow rate with a 21 min gradient. The heated capillary temperature was set to 275°C, the spray voltage to 2300 V and the funnel radiofrequency to 40. The mass spectrometer was operated in a data-independent mode (DIA) with a full MS range from 350 to 1650 at a resolution of 60000 at 200 m/z. The Automatic Gain Control (AGC) target was set to 300% with an injection time of 28 ms. The AGC value of the targeted MS2 experiment was set to 1000%. Twenty-two windows of variable sizes were defined for target MS2 (tMS2) acquisition and subjected to high-energy collisional dissociation (HCD) fragmentation with a normalized collision energy at 30%. Each tMS2 scan was acquired at a resolution of 30000 with a maximum ion injection time (IT) of 28 ms for a scan range of m/z 349.5 to 1650.5.

##### MS data analysis

The MS raw files were processed with Spectronaut version 15 (Biognosys, Zurich, Switzerland). A previously generated plasma spectral library was imported from (MaxQuant software analyses). The library contained 2137 protein groups and 16254 peptides. DIA files were searched against the library using default parameters except for the normalization which was set to local. Dynamic mass and retention time tolerances (for both MS1 and MS2) were applied. Qvalue cutoff was set to 1% both at precursor and protein level using a mutated decoy method. The calibration was performed based on a local regression model.

##### Bioinformatic analysis

Proteins data were exported from Spectronaut and loaded into the clinical knowledge graph (CKG)^77^ together with their matching experimental and clinical data. A stringent filter for missingness was applied (>70% completeness with each group), data was log-transformed and missing values were imputed by drawing random samples from a downshifted normal distribution (1.8 standard deviations from the mean with a standard deviation of 0.3 relative to the abundance distribution of all proteins in the sample).

### Quantification and statistical analysis

Baseline demographics were reported as absolute numbers with percentages (%) or medians with IQR. Comparison between the cohorts was performed using Fisher’s-exact test or Mann-Whitney U test as appropiate. Kolmogorov-Smirnov, Anderson–Darling, and Shapiro-Wilk normality tests were performed for all measured complement molecules and activation products. Due to nonparametric distribution, continuous data were analyzed using nonparametric tests or log-transformed to the base of 2. Correlations were performed using Spearman’s rank correlation test. ORs with 95% CIs were estimated using Logistic Regression analysis. Plasma proteins with significantly different levels between the two populations were identified by CKG analysis. In short, unpaired t-tests were computed and multiple hypothesis correction was applied using the Benjamini-Hochberg correction, only adjusted *p*-values <0.05 were considered statistically significant. Volcano plots were used to visualize plasma proteins with significant different protein levels between groups. To identify the enrichment of biological processes, GOBP enrichment analysis was performed on the set of significantly regulated proteins compared to the background of all quantified proteins in the proteomics experiment. Subsequently, a Logistic Regression prediction model, as implemented by Scikit-learn (random_state = 0, max_iter = 10000, class_weight = ”balanced”),^78^ was developed and trained on the DC and validated in the VC seperately. The features used in the prediction models were the list of differentially expressed proteins for 30- and 90-day mortality found in the analysis of the DC solely. Only sex and age matched samples from the DC were used for training, and an equal ratio of survivor and non-survivor patient samples were used in each cross-validation (CV) split (stratified k-fold splitting, number of CV = 5). Model performance on training data was assessed by the ROC AUC for the test set in each CV split. The AUC of the test sets from each CV were used to compute a ROC curve with confidence intervals. An average AUC was calculated across all splits, and reported together with the 95% CI and standard deviation. The final model was trained on the full balanced training set (*n* = 40) and applied to predict outcome for the VC samples. Based on the VC result, an AUC and confusion matrix was calculated (Table S4).

Statistical significance was defined as a *p*-value below 0.05 and denoted with asterisks as follows: non-significant (ns) for *p* > 0.05; ∗ for *p* ≤ 0.05; ∗∗ for *p* ≤ 0.01; ∗∗∗ for *p* ≤ 0.001; and ∗∗∗∗ for *p* ≤ 0.0001. Statistical analyses of complement molecules and activation products were performed using GraphPad Prism version 9.3.1 (GraphPad Software, San Diego, CA, USA) and R version 4.1.0 (R Foundation for Statistical Computing, Vienna, Austria).
