# Supplementary material for: Differences in biomarker levels and proteomic survival prediction across two COVID-19 cohorts with distinct treatments
Source: iScience. 2025 Feb 17;28(3):112046. doi: 10.1016/j.isci.2025.112046 (PMC11927729; doi:10.1016/j.isci.2025.112046)
Supplement: Document S1. Figures S1–S5 and Tables S1–S4 [file mmc1.pdf]

iScience, Volume *28*

Supplemental information

Differences in biomarker levels and proteomic survival prediction across two COVID-19 cohorts with distinct treatments

Cecilie Bo Hansen, Maria Elizabeth Engel Møller, Laura Pérez-Alós, Simone Bastrup Israelsen, Lylia Drici, Maud Eline Ottenheijm, Annelaura Bach Nielsen, Nicolai J. Wewer Albrechtsen, Thomas Benfield, and Peter Garred

Supplementary Figure 1. Scatter dot plots of biomarker levels stratified by hypertension status in the derivation cohort (DC) and validation cohort (VC).

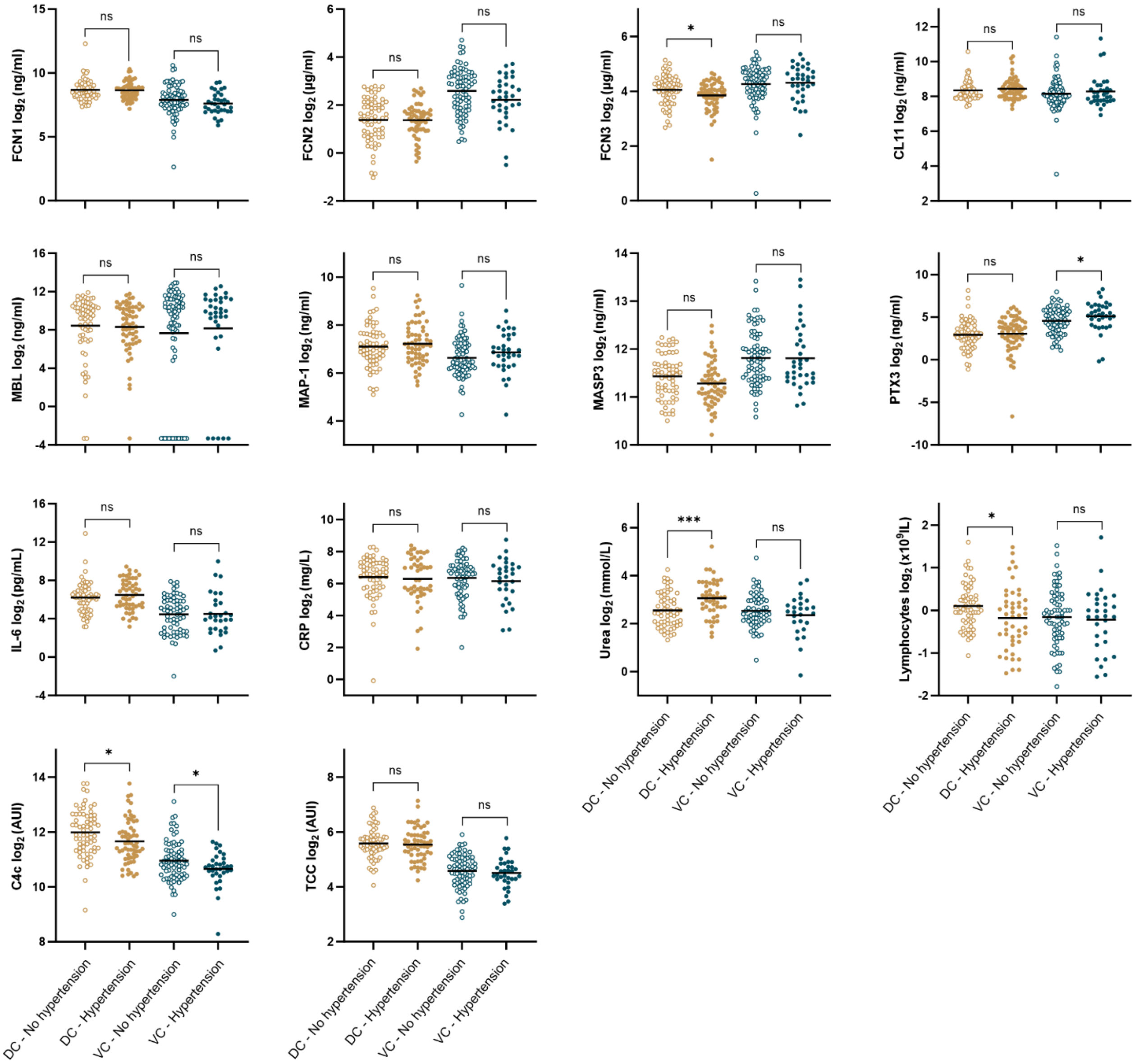

Yellow hollow dots represent DC patients without hypertension, yellow solid dots represent DC patients with hypertension, blue hollow dots represent VC patients without hypertension, and blue solid dots represent VC patients with hypertension. Horizontal black lines represent the mean. Differences between having hypertension and no hypertension were tested using an unpaired t-test. Non-significant, ns = p> 0.05; * = p≤ 0.05; ** = p≤ 0.01; *** = p≤ 0.001; **** = p≤ 0.0001.

Supplementary Figure 2. Scatter dot plots of biomarker levels stratified by <u>cardiovascular disease</u> status in the derivation cohort (DC) and validation cohort (VC).

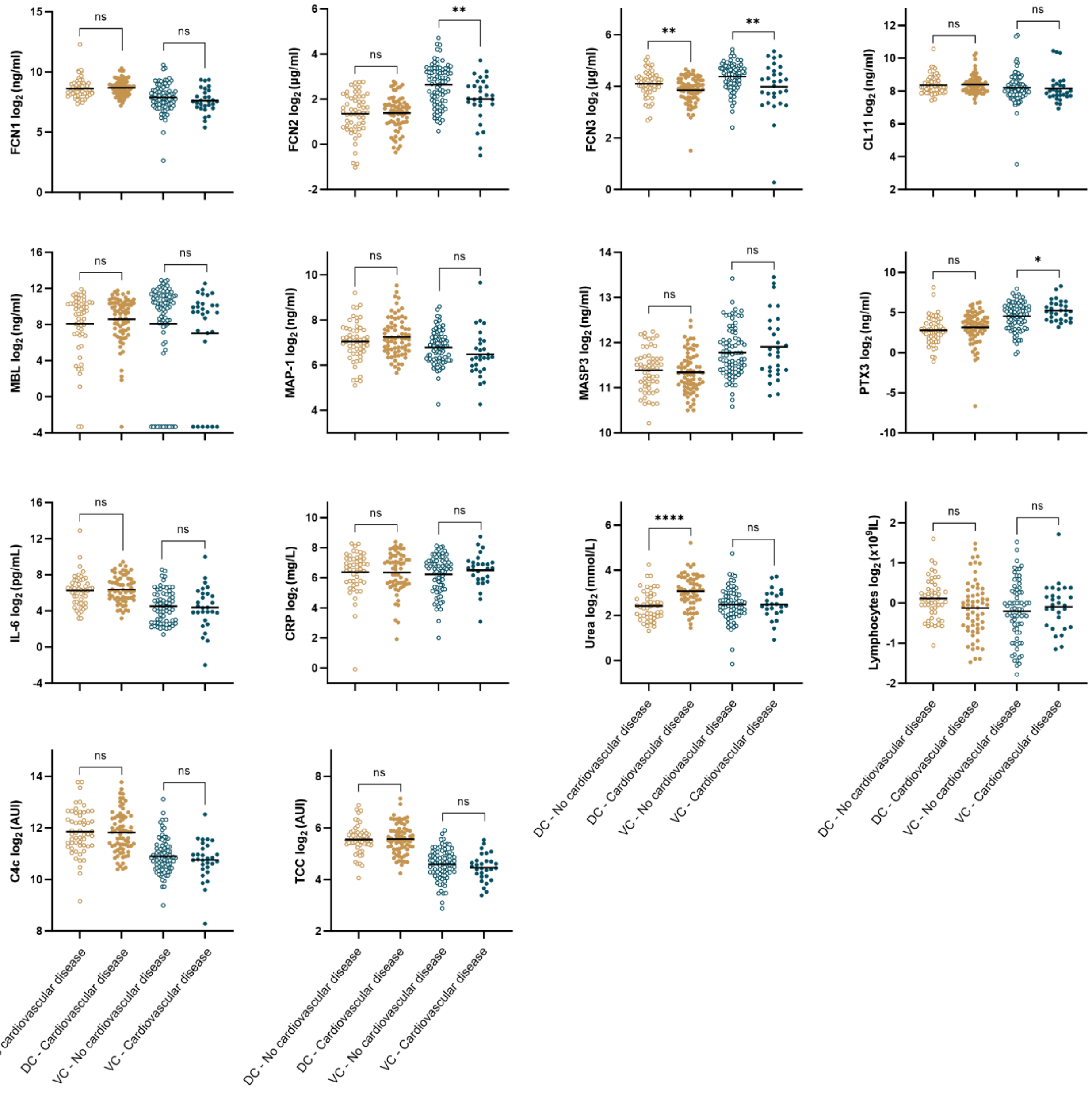

Yellow hollow dots represent DC patients without cardiovascular disease, yellow solid dots represent DC patients with cardiovascular disease, blue hollow dots represent VC patients without cardiovascular disease, and blue solid dots represent VC patients with cardiovascular disease. Horizontal black lines represent the mean. Differences between having cardiovascular disease and no cardiovascular disease were tested using an unpaired t-test. Non-significant, ns = p> 0.05; * = p≤ 0.05; ** = p≤ 0.01; *** = p≤ 0.001; **** = p≤ 0.0001.

Supplementary Figure 3. Scatter dot plots of biomarker levels stratified by <u>sex</u> in the derivation cohort (DC) and validation cohort (VC).

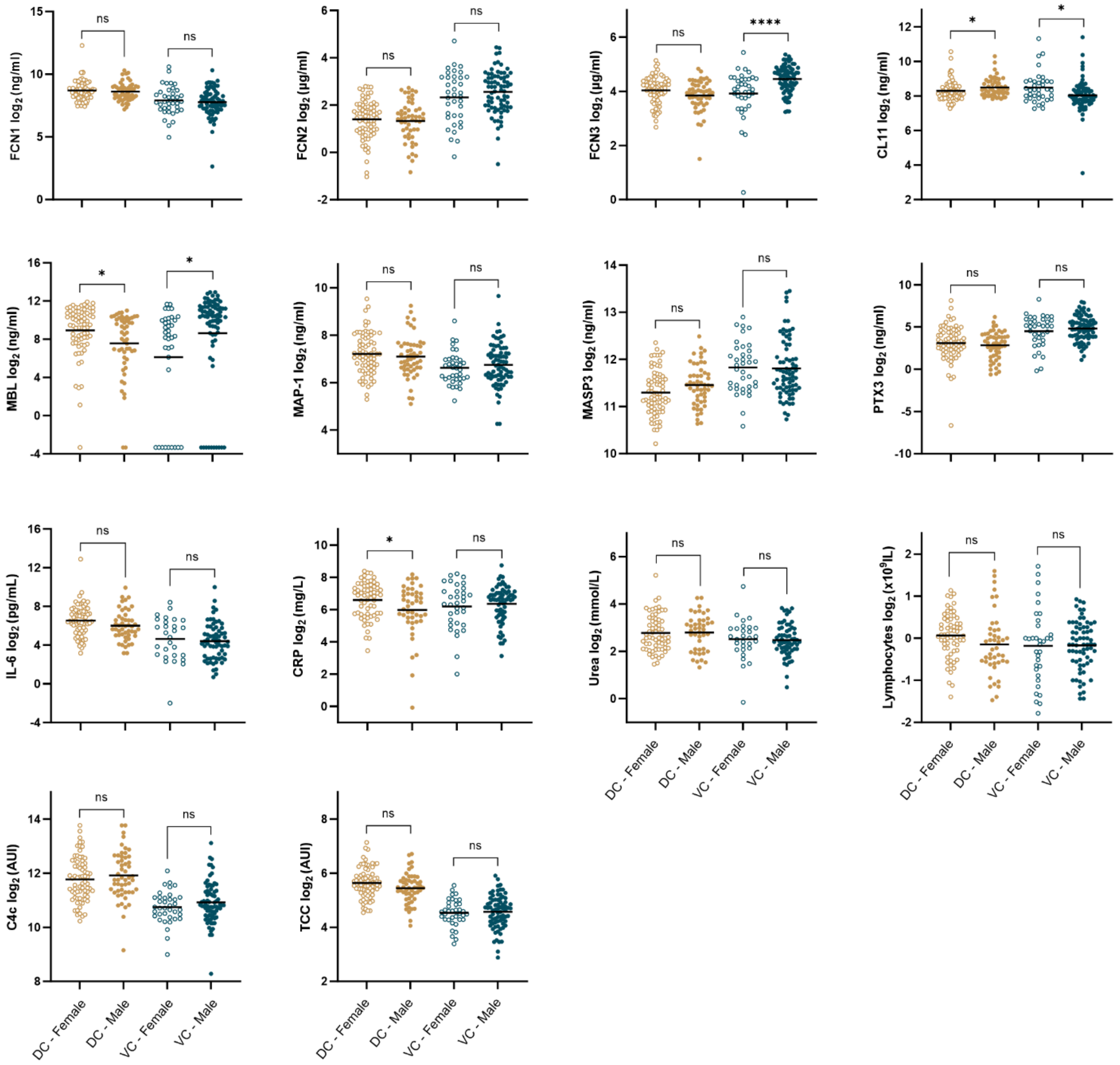

Yellow hollow dots represent DC females, yellow solid dots represent DC males, blue hollow dots represent VC females, and blue solid dots represent VC males. Horizontal black lines represent the mean. Differences between females and males were tested using an unpaired t-test. Non-significant, ns = p> 0.05; * = p≤ 0.05; ** = p≤ 0.01; *** = p≤ 0.001; **** = p≤ 0.0001.

Supplementary Figure 4. Proteomics analysis according to 90-day mortality

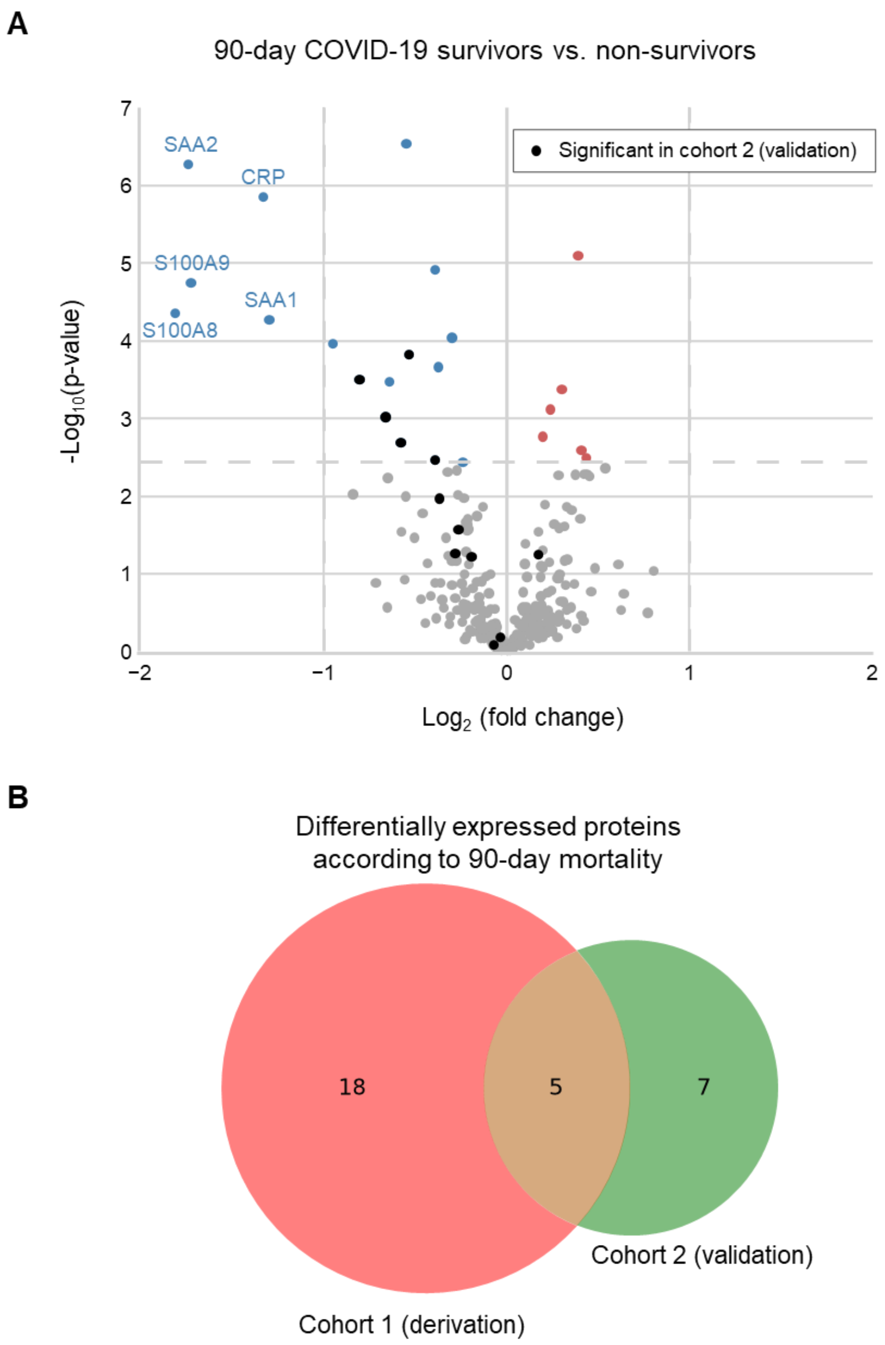

A. Volcano plot depicting the fold change and associated p-values of proteins in the derivation cohort (cohort 1) in 90-day survivors versus non-survivors. Significant DEPs in the validation cohort (cohort 2) are presented as black dots. **B**. Figure showing the number and overlap of DEPs between cohort 1 and cohort 2 according to 90-day mortality. The 5 overlapping DEPs: beta 2-microglobulin (B2M), leucine-rich alpha-2-glycoprotein 1 (LRG1), protein Z-dependent protease inhibitor (SERPINA10), CD44 and lymphatic vessel endothelial hyaluronan receptor 1 (LYVE1).

Supplementary Figure 5. Gene ontology biological processes enrichment analysis of proteins in the derivation cohort (cohort 1).

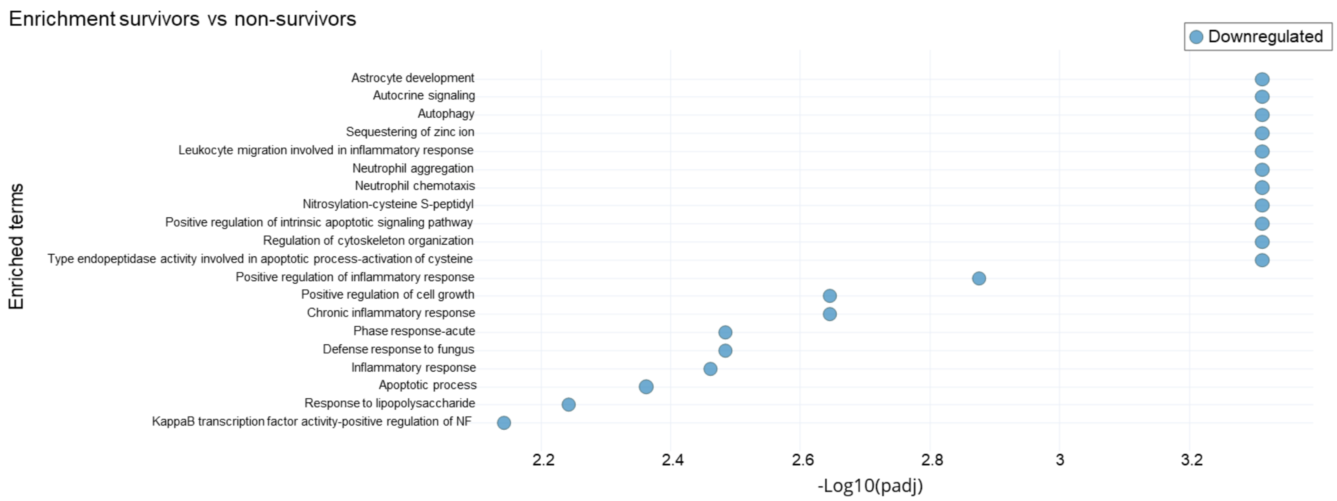

Supplementary Table 1. Spearman's rank correlation between age and biomarker levels

		**Derivation cohort** (n = 126)	
	r	95% confidence interval	*p* (two-tailed)
**Ficolin-1**	-0.1448	-0.3165 to 0.03610	0.1057
**Ficolin-2**	-0.3025	-0.4575 to -0.1296	*0.0006*
**Ficolin-3**	-0.3925	-0.5347 to -0.2287	*<0.0001*
**MBL**	-0.1354	-0.3079 to 0.04568	0.1306
**PTX3**	0.222	0.04373 to 0.3865	*0.0125*
**MAP-1**	-0.05759	-0.2351 to 0.1237	0.5219
**MASP-3**	-0.1551	-0.3260 to 0.02558	0.0829
**Collectin-11**	-0.01751	-0.1969 to 0.1630	0.8457
**C4c**	-0.103	-0.2778 to 0.07840	0.251
**TCC**	-0.2131	-0.3785 to -0.03443	*0.0166*
**IL-6**	0.2881	0.1063 to 0.4513	*0.0017*
**CRP**	-0.06347	-0.2547 to 0.1326	0.514
**Urea**	0.5625	0.4125 to 0.6828	*<0.0001*
**Lymphocyte count**	-0.1607	-0.3444 to 0.03481	0.0966
		**Validation cohort** (n = 112)	
**Ficolin-1**	-0.1042	-0.2893 to 0.08847	0.2743
**Ficolin-2**	-0.3891	-0.5399 to -0.2140	*<0.0001*
**Ficolin-3**	-0.3048	-0.4684 to -0.1209	*0.0011*
**MBL**	-0.1946	-0.3717 to -0.003785	*0.0398*
**PTX3**	0.1866	-0.004426 to 0.3646	*0.0488*
**MAP-1**	-0.1415	-0.3237 to 0.05077	0.1367
**MASP-3**	-0.2238	-0.3977 to -0.03440	*0.0177*
**Collectin-11**	-0.02553	-0.2154 to 0.1662	0.7893
**C4c**	-0.06209	-0.2500 to 0.1304	0.5155
**TCC**	-0.2053	-0.3813 to -0.01500	*0.0299*
**IL-6**	0.07098	-0.1407 to 0.2764	0.4989
**CRP**	-0.05082	-0.2493 to 0.1518	0.6137
**Urea**	0.06065	-0.1532 to 0.2690	0.568
**Lymphocyte count**	0.0562	-0.1455 to 0.2534	0.5748

Supplementary Table 2. Logistic regression analysis of biomarkers association with 30-day mortality in the derivation and validation cohorts.

	**Derivation cohort** (n = 126)		
	Crude OR [95% CI]	Adjusted OR (95% CI)[1]	*p*
**Ficolin-1, per doubling**	1.72 [1.01-2.92]	2.30 [1.16-4.53]	*0.016*
**Ficolin-2, per doubling**	0.90 [0.56-1.45]	1.20 [0.67-2.17]	0.537
**Ficolin-3, per doubling**	0.64 [0.31-1.33]	1.25 [0.51-3.07]	0.627
**MBL, per doubling**	1.01 [0.88-1.18]	1.05 [0.87-1.26]	0.617
**PTX3, per doubling**	2.61 [1.73-3.92]	2.48 [1.61-3.84]	*<0.001*
**MAP-1, per doubling**	2.13 [1.29-3.52]	3.01 [1.60-5.63]	*<0.001*
**MASP-3, per doubling**	1.03 [0.43-2.44]	1.88 [0.65-5.37]	0.241
**Collectin-11, per doubling**	1.37 [0.69-2.70]	1.50 [0.70-3.25]	0.301
**C4c, per doubling**	1.52 [0.93-2.47]	2.24 [1.24-4.05]	*0.008*
**TCC, per doubling**	1.28 [0.63-2.59]	1.88 [0.82-4.26]	0.134
**IL-6, per doubling**	2.32 [1.59-3.39]	2.29 [1.49-3.54]	*<0.001*
**CRP, per doubling**	1.29 [0.90-1.86]	1.32 [0.85-2.05]	0.212
**P-Urea, per doubling**	4.25 [2.06-8.77]	3.10 [1.31-7.36]	*0.010*
**B-lymphocyte count, per doubling**	0.47 [0.22-0.97]	0.50 [0.21-1.17]	0.110
**Anti-RBD IgG, per doubling**	0.92 [0.84-1.01]	0.95 [0.86-1.06]	0.378
**Anti-RBD IgM, per doubling**	0.95 [0.87-1.03]	1.00 [0.90-1.10]	0.924
**Anti-RBD IgA, per doubling**	0.95 [0.83-1.10]	0.98 [0.84-1.14]	0.830
	**Validation cohort** (n = 112)		
	Crude OR (95% CI)	Adjusted OR (95% CI)[1]	*p*
**Ficolin-1, per doubling**	0.79 [0.50-1.25]	0.82 [0.51-1.35]	0.444
**Ficolin-2, per doubling**	0.60 [0.35-1.05]	0.78 [0.42-1.45]	0.430
**Ficolin-3, per doubling**	0.54 [0.27-1.07]	0.51 [0.22-1.17]	0.110
**MBL, per doubling**	0.90 [0.63-1.27]	0.89 [0.58-1.38]	0.612
**PTX3, per doubling**	1.78 [1.15-2.76]	1.74 [1.11-2.75]	*0.016*
**MAP-1, per doubling**	0.53 [0.27-1.08]	0.52 [0.24-1.10]	0.087
**MASP-3, per doubling**	0.89 [0.35-2.26]	1.08 [0.43-2.70]	0.868
**Collectin-11, per doubling**	1.87 [1.05-3.32]	2.11 [1.14-3.92]	*0.018*
**C4c, per doubling**	0.62 [0.28-1.37]	0.58 [0.24-1.43]	0.238
**TCC, per doubling**	0.51 [0.20-1.31]	0.56 [0.20-1.61]	0.283
**IL-6, per doubling**	0.72 [0.52-1.00]	0.73 [0.52-1.02]	0.062
**CRP, per doubling**	0.84 [0.54-1.32]	0.79 [0.49-1.28]	0.349
**P-Urea, per doubling**	0.93 [0.43-2.02]	0.90 [0.40-2.05]	0.806
**B-lymphocyte count, per doubling**	1.34 [0.59-3.05]	1.35 [0.58-3.16]	0.492
**Anti-RBD IgG, per doubling**	0.85 [0.74-0.99]	0.89 [0.76-1.04]	0.141
**Anti-RBD IgM, per doubling**	0.87 [0.70-1.08]	0.95 [0.75-1.21]	0.667
**Anti-RBD IgA, per doubling**	0.84 [0.71-0.98]	0.85 [0.73-1.01]	0.060

OR: Odds ratio, CI: confidence interval, MBL:, TCC:, CRP: C-reactive protein, IL-6: interleukin-6, RBD:
[1]Adjusted for age, sex, hypertension, and cardiovascular disease.

Supplementary Table 3. Logistic regression analysis of biomarkers association with 90-day mortality in the derivation and validation cohorts.

	**Derivation cohort** (n = 126)		
	Crude OR [95% CI]	Adjusted OR (95% CI)[1]	*p*
**Ficolin-1, per doubling**	2.10 [1.22-3.64]	3.48 [1.61-7.50]	*0.001*
**Ficolin-2, per doubling**	0.88 [0.56-1.39]	1.19 [0.68-2.06]	0.544
**Ficolin-3, per doubling**	0.60 [0.30-1.22]	1.09 [0.47-2.52]	0.848
**MBL, per doubling**	1.04 [0.90-1.20]	1.10 [0.92-1.31]	0.310
**PTX3, per doubling**	2.35 [1.63-3.39]	2.28 [1.55-3.37]	*<0.001*
**MAP-1, per doubling**	1.86 [1.17-2.98]	2.48 [1.40-4.39]	*0.002*
**MASP-3, per doubling**	0.86 [0.38-1.97]	1.44 [0.54-3.83]	0.462
**Collectin-11, per doubling**	1.20 [0.62-2.31]	1.22 [0.59-2.55]	0.590
**C4c, per doubling**	1.06 [0.67-1.66]	1.33 [0.79-2.25]	0.286
**TCC, per doubling**	1.54 [0.78-3.04]	2.47 [1.10-5.55]	*0.028*
**IL-6, per doubling**	2.46 [1.69-3.58]	2.52 [1.63-3.91]	*<0.001*
**CRP, per doubling**	1.35 [0.94-1.93]	1.45 [0.95-2.22]	0.081
**P-Urea, per doubling**	4.08 [2.03-8.20]	2.96 [1.32-6.62]	*0.008*
**B-lymphocyte count, per doubling**	0.52 [0.26-1.04]	0.60 [0.28-1.28]	0.185
**Anti-RBD IgG, per doubling**	0.92 [0.84-1.00]	0.95 [0.86-1.05]	0.309
**Anti-RBD IgM, per doubling**	0.93 [0.86-1.01]	0.97 [0.88-1.07]	0.578
**Anti-RBD IgA, per doubling**	0.93 [0.81-1.06]	0.96 [0.83-1.11]	0.590
	**Validation cohort** (n = 112)		
	Crude OR (95% CI)	Adjusted OR (95% CI)[1]	*p*
**Ficolin-1, per doubling**	0.83 [0.53-1.30]	0.89 [0.54-1.45]	0.632
**Ficolin-2, per doubling**	0.56 [0.33-0.97]	0.72 [0.39-1.33]	0.294
**Ficolin-3, per doubling**	0.48 [0.24-0.98]	0.44 [0.19-1.00]	*0.049*
**MBL, per doubling**	0.95 [0.67-1.35]	0.96 [0.63-1.48]	0.864
**PTX3, per doubling**	1.76 [1.15-2.68]	1.68 [1.08-2.60]	*0.021*
**MAP-1, per doubling**	0.58 [0.29-1.14]	0.57 [0.28-1.17]	0.126
**MASP-3, per doubling**	0.83 [0.33-2.09]	0.98 [0.40-2.39]	0.956
**Collectin-11, per doubling**	1.75 [1.00-3.08]	1.98 [1.08-3.63]	*0.028*
**C4c, per doubling**	0.65 [0.30-1.40]	0.65 [0.27-1.53]	0.321
**TCC, per doubling**	0.64 [0.26-1.59]	0.74 [0.26-2.08]	0.572
**IL-6, per doubling**	0.73 [0.53-1.00]	0.74 [0.53-1.01]	0.061
**CRP, per doubling**	0.88 [0.57-1.36]	0.83 [0.52-1.33]	0.434
**P-Urea, per doubling**	0.90 [0.52-2.39]	1.14 [0.51-2.56]	0.753
**B-lymphocyte count, per doubling**	1.39 [0.62-3.09]	1.39 [0.60-3.22]	0.439
**Anti-RBD IgG, per doubling**	0.86 [0.74-0.99]	0.89 [0.77-1.04]	0.146
**Anti-RBD IgM, per doubling**	0.85 [0.69-1.05]	0.92 [0.73-1.17]	0.507
**Anti-RBD IgA, per doubling**	0.82 [0.70-0.96]	0.85 [0.72-0.99]	*0.043*

OR: Odds ratio, CI: confidence interval, MBL:, TCC:, CRP: C-reactive protein, IL-6: interleukin-6, RBD:
[1]Adjusted for age, sex, hypertension, and cardiovascular disease.

Supplementary Table 4. Confusion matrices for 30- and 90-day prediction models

**30-day**		*Predicted*	
		Non-survivor	Survivor
Observed	Non-survivor	11	6
	Survivor	32	86

**90-day**		*Predicted*	
		Non-survivor	Survivor
Observed	Non-survivor	14	5
	Survivor	28	88

	**30-day**	**90-day**
**Accuracy:**	0.7185	0.7556
**Precision:**	0.9348	0.9462
**Recall:**	0.7288	0.7586
**ROC AUC:**	0.7453	0.8004
**F1:**	0.8190	0.8421
**Matthews:**	0.2677	0.3722
